# Glia Talk Back

**DOI:** 10.1371/journal.pbio.1001990

**Published:** 2014-11-11

**Authors:** Rachel Jones

**Affiliations:** Freelance Science Writer and Editor, Welwyn, Hertfordshire, United Kingdom

The cells in mammalian brains fall into two main categories: neurons and glia. The idea that glia are second-class citizens in the brain, existing merely to sustain and support the more important and complex neurons, has been challenged in recent years by evidence that glial cells have important functions of their own. Perhaps the best-studied glial cells from this point of view are astrocytes, which outnumber neurons five-to-one and are involved in synaptic transmission and the regulation of neuronal processing.

Another type of glia, oligodendrocytes, produces the myelin sheaths that provide insulation for the brain's wiring—neuronal axons—and they do so in response to neuronal activity. The cells that give rise to oligodendrocytes (oligodendrocyte precursor cells, or OPCs) also seem to have additional functions, including being the only type of glia to receive synaptic inputs from neurons. Another hint that OPCs are important for more than just producing oligodendrocytes is that they are found throughout the brain in numbers far greater than would be needed for that role.

OPCs have a membrane-spanning signaling protein called NG2 (for neuron/glia antigen 2) that is not found on any other glia or on neurons. This protein, along with the synapses between neurons and OPCs, disappears when OPCs differentiate into oligodendrocytes. OPCs change their behavior in response to neuronal activity, but exactly how they do so—and whether this communication is unidirectional or goes both ways—is unclear.

A new study by Dominik Sakry, Angela Neitz, et al., published in *PLOS Biology*, has investigated the role of NG2 in the communication between neurons and OPCs. Previous results showed that it was possible to extract an extracellular part of NG2 from the extracellular matrix, indicating that it was shed from the protein. Now, Sakry et al. have shown that this fragment is produced by activity-dependent cleavage of NG2 by proteins called secretases.

**Figure 1:**
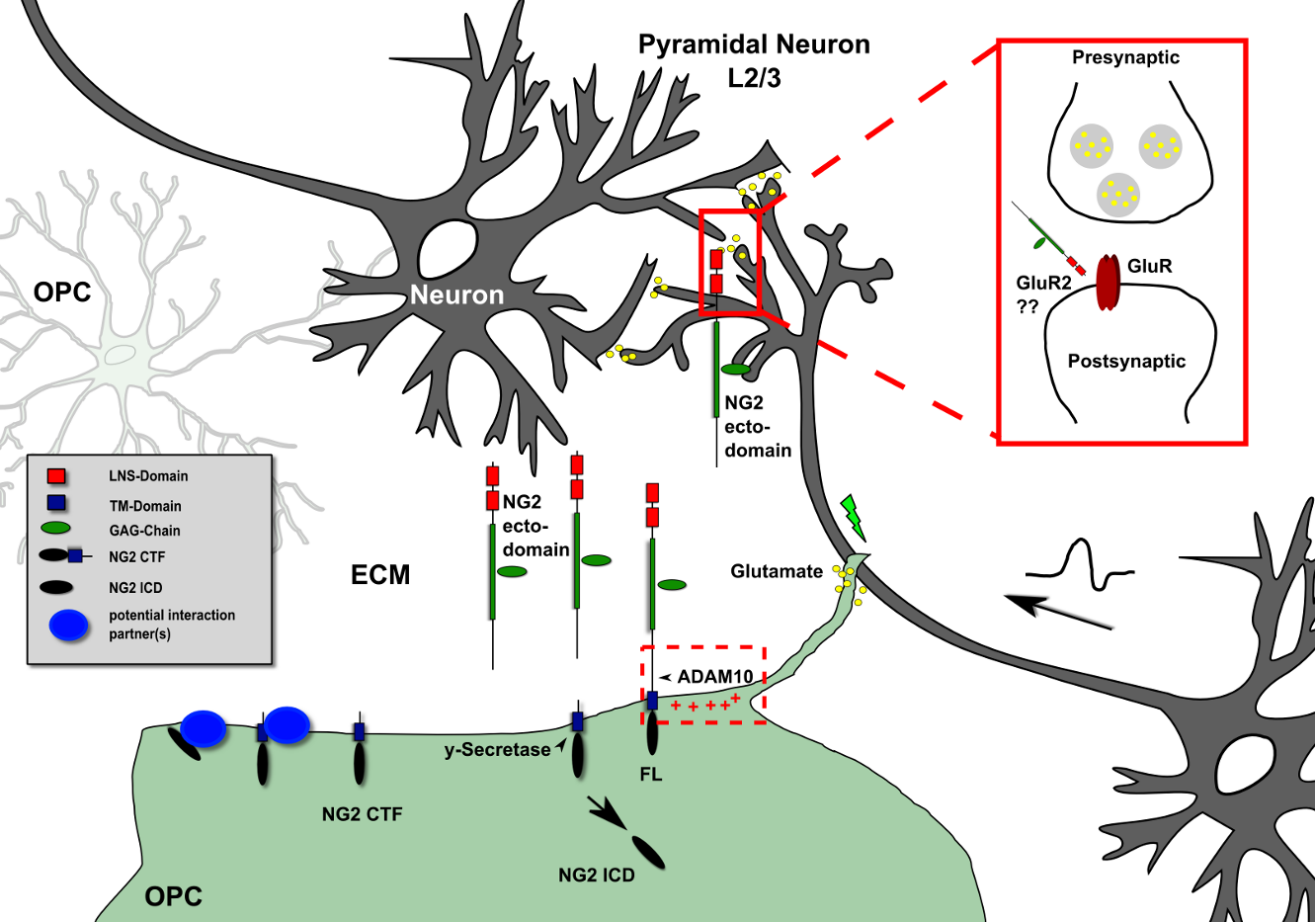
Oligodendrocyte precursor cells (OPC, green) are integrated into the neuronal network (gray) of the mammalian cortex. Activity-dependent extracellular NG2 protein cleavage releases an ectodomain into the extracellular matrix (ECM). The LNS domains on the ectodomain influence postsynaptic glutamatergic signal transduction of neuronal L4-L2/3 innervations of pyramidal neurons of the somatosensory cortex. Within this network, the presence of the NG2 LNS domains causes an alteration of AMPA receptor (GluR) kinetics, suggesting modulation of the levels of GluR2-containing AMPA receptors at the cell surface.

The new results demonstrate that a secretase called ADAM10, together with other secretases, cleaves NG2 on cultured OPCs in response to increases in neuronal network activity (Figure 1). This process generates three fragments of NG2: a large ectodomain, which is released extracellularly, and two smaller pieces, one of which remains attached to the membrane and the other of which is released inside the cell. When the researchers used a specific inhibitor to block the activity of ADAM10, both activity-dependent and constitutive cleavage of NG2 were prevented.

To investigate the physiological functions of NG2 cleavage in the brain, the researchers used mutant mice that lacked NG2. In the brain's somatosensory cortex, neurons called pyramidal neurons usually show a strong and lasting increase in synaptic strength in response to certain patterns of activity that is called long-term potentiation (LTP) and is crucially involved in memory and learning; this LTP was severely impaired in the mutant mice and also in normal mice in which ADAM10 was inhibited.

But what was the molecular basis of these changes? Neurotransmitter receptors such as AMPA receptors consist of complexes of protein subunits, and the functional properties of these receptors depend on the combination of subunits that makes up each receptor. In mice lacking NG2, neuronal AMPA receptors showed altered subunit compositions compared with receptors in normal mice, probably accounting for the reduced receptor currents and impaired LTP.

Remarkably, when cultured brain slices from mice that lack NG2 were treated with a recombinant protein containing the two neurexin-like domains from the extracellular domain of NG2 that is shed by cleavage, the properties of neuronal AMPA receptors in the slices returned to normal. The neurexin-like domains therefore seem to be crucial for the modulation of neuronal physiology by NG2 cleavage. Finally, mutant mice lacking NG2 showed deficits in behaviors that depend on the somatosensory cortex, highlighting the physiological relevance of this bi-directional communication between neurons and OPCs.

Although many questions are raised by these results, they nonetheless show that OPCs not only receive input from neurons, but also can modulate neuronal properties and activity. These findings add weight to the concept that glial cells are far more than simple support cells—the more we look, the more functions we find for these jacks-of-all-trades. It is becoming clear that neurons and glia talk to each other constantly; further details of this conversation can only help us to understand the mysteries of the brain.

**Sakry D, Neitz A, Singh J, Frischknecht R, Marongiu D, et al. (2014) Oligodendrocyte Precursor Cells Modulate the Neuronal Network by Activity-Dependent Ectodomain Cleavage of Glial NG2.** doi:10.1371/journal.pbio.1001993

